# Overview of the pre-clinical and clinical studies about the use of CAR-T cell therapy of cancer combined with oncolytic viruses

**DOI:** 10.1186/s12957-021-02486-x

**Published:** 2022-01-13

**Authors:** Ali Zarezadeh Mehrabadi, Fatemeh Roozbahani, Reza Ranjbar, Mahdieh Farzanehpour, Alireza Shahriary, Ruhollah Dorostkar, Hadi Esmaeili Gouvarchin Ghaleh

**Affiliations:** 1 Applied Virology Research Center, Baqiyatallah University of Medical Sciences, Tehran, Iran; 2 Immunology Department, School of Medicine, Iran University of Medical Sciences, Tehran, Iran; 3 Department of Microbiology and Virology, Faculty of Medicine, Mazandaran University of Medical Sciences, Sari, Iran; 4 Molecular Biology Research Center, Systems Biology and Poisonings Institute, Baqiyatallah University of Medical Sciences, Tehran, Iran; 5 Chemical Injuries Research Center, Systems Biology and Poisonings Institute, Baqiyatallah University of Medical Sciences, Tehran, Iran

**Keywords:** Chimeric antigen receptor T cells (CAR T cells), Combined approaches, Oncolytic viruses, Cancers

## Abstract

**Background:**

Cancer is one of the critical issues of the global health system with a high mortality rate even with the available therapies, so using novel therapeutic approaches to reduce the mortality rate and increase the quality of life is sensed more than ever.

**Main body:**

CAR-T cell therapy and oncolytic viruses are innovative cancer therapeutic approaches with fewer complications than common treatments such as chemotherapy and radiotherapy and significantly improve the quality of life. Oncolytic viruses can selectively proliferate in the cancer cells and destroy them. The specificity of oncolytic viruses potentially maintains the normal cells and tissues intact. T-cells are genetically manipulated and armed against the specific antigens of the tumor cells in CAR-T cell therapy. Eventually, they are returned to the body and act against the tumor cells. Nowadays, virology and oncology researchers intend to improve the efficacy of immunotherapy by utilizing CAR-T cells in combination with oncolytic viruses.

**Conclusion:**

Using CAR-T cells along with oncolytic viruses can enhance the efficacy of CAR-T cell therapy in destroying the solid tumors, increasing the permeability of the tumor cells for T-cells, reducing the disturbing effects of the immune system, and increasing the success chance in the treatment of this hazardous disease.

In recent years, significant progress has been achieved in using oncolytic viruses alone and in combination with other therapeutic approaches such as CAR-T cell therapy in pre-clinical and clinical investigations. This principle necessitates a deeper consideration of these treatment strategies. This review intends to curtly investigate each of these therapeutic methods, lonely and in combination form. We will also point to the pre-clinical and clinical studies about the use of CAR-T cell therapy combined with oncolytic viruses.

## Background

*Cancer* is a lethal disease discriminated by long-term inflammation [1]. Cancer impacted almost 19 million patients in 2020, and 10 million people died due to it. Thus, developing a stable infrastructure for preventing and caring activities is indispensable for this world health issue [2].

Cancer treatment involves a wide range of modalities, including chemotherapy, radiotherapy, surgery, and medications such as monoclonal antibodies [3]. Additionally, physicians and researchers in this field are now looking for new approaches with higher efficacy, specificity, and fewer complications. Adoptive cell transfer (ACT) and oncolytic viruses are two novel therapeutic methods; several clinical trials are being conducted regarding these modalities.

Engineered T-cells expressing chimeric antigen receptors (CARs) specific to cancer cells have recently attracted much attention. CARs are recombinant receptors typically targeting surface molecules (here, cancer cell surface molecules) [4]. Generally, CARs include three main components: an extracellular antigen recognition domain of the single-chain fragment variant (scFv) region, a transmembrane domain, and an intracellular CD3ζ domain [5]. Novel CAR constructs evolve by adding co-stimulatory domains or targeting domains [6]. Autologous CAR-T cells can attack cancer cells and destroy them specifically.

CAR-T cell therapy is more effective in treating hematologic malignancies than solid tumors. For example, using CAR-T cells expressing anti-CD19 antigens (PAN B cell marker) has successfully treated acute lymphoblastic B-cell cancer of the children, non-Hodgkin lymphoma, and chronic lymphocytic leukemia [7].

The concept of immunotherapy in cancer has attracted the attention of physicians for centuries; thus, the relationship between microbial infection and mediated spontaneous tumor inhibition has been frequently assessed and discussed [8]. In the seventeenth to nineteenth centuries, different types of immunotherapy have been widely used. Two critical instances are using septic bandages for ulcerative tumor treatment and leaving surgical wounds open deliberately to accelerate wound healing [9]. According to the medical records of William Cole, the surgeon, he has treated cancer patients with bacterial lysates [10]. Oncolytic viruses selectively attack cancer cells, infect and lyse them, though they do not infect healthy cells. The oncolytic viruses are either wild viruses or laboratory-modified wild viruses. By using biotechnological approaches, a new era of viral-based therapies has started to cause fewer complications for the patients [11, 12]. In addition to oncolytic activity, OVs are very effective in stimulating inflammation and immune response against themselves and the tumor cells. However, the outcome of the immune response is associated with some complexities; the OV-mediated anti-tumor immunity is eventually effective [10, 13]. Many OVs act like vaccines and lead to robust and specific TCD8 + -mediated anti-tumor reactions, which are frequently associated with the formation of significant memory cells [14, 15]. Conclusively, OVs may turn into an effective therapeutic modality in treating various types of tumors soon. In cancer treatment, *gene therapy* is defined as using genetic molecules to manipulate target cells and tissues to treat cancer patients [16]. Two types of gene therapy modalities have been proven effective in recent years, including using CAR-T cells and oncolytic viruses for tumor treatment. Oncolytic viruses are independently ineffective in the treatment of cancers, especially giant and metastatic tumors. Hence, oncolytic viruses can be used in combination with other treatments [17], including immune checkpoint blockers [18] and CAR-T cell therapy [19]. The combination of oncolytic viruses and CAR-T cells can be considered a new complementary strategy to overcome the limitations of using each of these therapies alone. This review aims to highlight the importance of CAR-T cells and oncolytic viruses as well as their use in monotherapy and combination therapy.

### Overview of oncolytic viruses

Viruses are intracellular infectious particles that rely on the host cells for survival and proliferation; viral particles lead to pathogenesis and inflammation in the host cells [20]. Viruses are composed of three main parts: Genome is the innermost part of the viral particles (RNA or DNA) surrounded and protected by a protein coating called a capsid. The outermost part of the viral particles is the lipid coating or envelope, which facilitates viral binding to the host cells. Oncolytic viruses have no difference, though DNA oncolytic viruses are superior to other viruses due to their more giant genome, high stability of their polymerase enzyme, genome homogeneity, and high proliferative ability [21]. RNA viruses are suitable for tumors of the central nervous system due to their small size and ability to cross the blood–brain barrier [22].

Using viruses in cancer therapy dates back to the early nineteenth century as the reported treatment of patients with leukemia following viral infections. In the 1950s and 1960s, our viral knowledge expanded acknowledgments to the significant advancements of cell culture systems. Viral therapy attracted particular attention, and many viruses such as hepatitis, West Nile, and Epstein-Barr virus were frequently used in cancer treatment. These studies provided valuable information despite the variable and controversial results [23–25]. Some of these studies provided promising results. For example, in 1956, 30 female patients with advanced epidermoid cancer of the cervix were treated with adenovirus. In most patients, tumor region necrosis was observed, which was limited to the cancer tissue leading to no damage to the healthy tissue [26]. In the 1970s and 1980s, using viruses as a cancer therapeutic method was neglected; two decades later, this therapeutic method re-emerged under the title of “oncolytic viruses” [24].

In recent years, virotherapy for cancer has remarkably progressed. Moreover, these viruses may be used for cancer treatment due to their unique characteristics. Talimogenge lahreparepvec [T-vec] was the first oncolytic virus identified and registered for treating irrecoverable metastatic melanoma in the USA in 2015 [27, 28]. This virus infected and hit tumor cells, stimulated macrophages, and dendritic cells with pattern recognition receptors (PRP) through pathogen-associated molecular patterns [PAMP] released from killed tumor cells [20, 29]. On the other hand, oncolytic viruses lead to DAMP production and activation of dendritic cells through tumor tissue destruction [30], and eventually, mature dendritic cells activate anti-tumor T cells by presenting tumor epitopes [31].

Some strategies such as using tumor-specific promotor and modification of viral protein may improve the oncolytic efficiency of the viruses, allowing the viral particles to merely infect tumor tissue without affecting healthy tissue [32]; moreover, antibodies, cytokines, and immune stimulants could be associated with the viral particles so that viral particles tolerate the tumor microenvironment and improve the efficacy [33].

Some oncolytic viruses, including parvoviruses, reoviruses, Newcastle disease virus, myxoma virus, Seneca Valley virus, and coxsackievirus, naturally have the ability of target cell recognition [34, 35]. These viruses proliferate after inserting the cells and releasing tumor-specific antigens, danger signals, interferon I production, and eventually tumor lysis and destruction [30]. They selectively enter their target cells (tumors) and do not infect healthy cells, though they induce immunity to other cells. On the other hand, some viruses, including adenovirus, herpes simplex virus, rubella virus, poliovirus, and vesicular stomatitis virus, do not inherit this feature and require genetic modification [34] (Table 1).

**Table 1:** A summary of the oncolytic viruses and their characteristics

**Features Viruses**	**Engineered viruses in studies**	**Particle size**	**Cell entry mechanism**	**Immunogenicity**	**Advantages**	**Disadvantages**
**Adenoviruses (dsDNA)**	1. ONYX‐015 in head and neck cancer	32 kb	Endocytosis	Low	• Can be controlled geneticall • Clinical trial encounter • Great information of viral protein work • Low pathogenicity	• Replication cannot be easily shut-off
	2.DNX‐2401 (delta‐24‐RGD) in ovarian cancer					
	3. CG0070 in nonmuscle invasive bladder cancer					
**Herpes simplex virus (dsDNA)**	1. T‐VEC (talimogene laherparepvec) in melanoma	152 kb	Endocytosis; penetration	Low	• Can be easily manipulated genetically • Clinical trial experience; drugs exist to shut-off unwanted viral replication	• Side impacts incorporate genuine or possibly lethal disease • Unknown activity of numerous HSV1 qualities
	2. HF10 in pancreatic cancer					
**Pox virus (vaccinia virus) (dsDNA)**	Pexa-Vec(JX-594) in primary hepatocellular carcinoma	130–375 kb	Membranepenetration and fusion	High	• Can be easily manipulated genetically • Clinical trial experience • Stable in human serum • Excellent human safety • Large capacity for encoding transgenes (50 kb) • Anti-tumor vascular activity	• Undesired viral replication cannot be easily shut-off
						• Unknown action of many genes • Side effects might include potentially fatal or seriously morbid disease
**Poliovirusss ( +) RNA**	PVS‐RIPO in recurrent glioblastoma	7.5 kb	Receptor-mediated endocytosis	Moderate	• Good knowledge of viral gene functions	• Cannot be easily manipulated genetically • No clinical trial experience • Viral replication cannot be easily shut-off • Associated with fatality or serious disease
**Measles** **virusss (–) RNA**	MV‐NIS in ovarian cancer	16 kb ~	Membrane fusion	Low	• Extensively studied • Easily manipulated • Genomic stability • No integration into host genome • Adjustable gene • Crossing of physiological membranes	• Preexisting immune response due to vaccination
**ReovirusesdsRNA**	RT3D (Reolysin®) in head and neck cancer	22–27 kb	Endocytosis	Low	• Associated with relatively mild diseases • Good knowledge of viral gene function • Growth advantage in human cells	• Cannot be easily manipulated genetically • No clinical trial experience • Viral replication cannot be easily shut-off

In 1999, immune response induction by oncolytic cells was recognized via oHSV for the first time. This virus acted as an “in situ cancer vaccine” and induced tumor-specific CTL and tumor cell death by the effector T cells against inoculated tumors [15].

Like gene therapy, desired genes for proper intracellular function may be added to oncolytic viruses to substitute viral genes [36]. One of the fantastic ways that can be used to kill tumors is to induce a “suicide gene” in tumor cells [37]. It is noteworthy that desired tumor-associated antigens may bond to the oncolytic viruses’ surface through electrostatic bindings, even without gene therapy or genetic engineering [38].

### Overview of CAR-T and CAR-NK cell therapies

In the last years, genetically modified immune cells, mainly T cells and natural killer (NK) cells that express chimeric antigen receptors (CARs), have had significant success in killing cancer cells [39]. CARs expressed on T or NK cells can enable them to detect a specific antigen expressed on tumor cells in the patient. CARs include three main components: an extracellular domain for tumor antigen recognition, a transmembrane domain, and one or more intracellular signaling domains leading to T-cell activation. The single-chain fragment variable (scFv) of a CAR, typically consists of variable heavy (VH) and variable light (VL) chains of an antibody which are bonded by a linker peptide SCFV, binds to an intracellular signaling molecule composed of a CD3ζ signaling domain or an intracellular signaling immunoreceptor tyrosine-based activation motif (ITAM). On the other hand, co-activator molecules such as CD28 and 4-1BB may be involved in the intracellular domain of the CAR. The main advantage of CAR-based methods for cancer immunotherapy is that SCFV shows a considerably higher affinity for antigen binding compared with TCR. Moreover, unlike TCR, SCFV acts in an MHC-independent manner [40, 41].

CAR T or NK cell therapy process comprises several steps that can take several weeks. First, T or NK cells are isolated from the patient’s or donor’s blood through leukapheresis, and thereupon genetically modified ex vivo, using viral or non-viral transfection methods. The CAR-modified immune cells are then grown and multiplied in the laboratory until sufficient cell numbers are produced. When the CAR T or NK cells are prepared, the patient receives a brief lymphodepleting chemotherapy course, followed by CAR T or NK cell infusion [42].

More than 450 clinical trials analyze CAR-T cells to treat cancer, especially hematological malignancies [42]. Among these clinical trials, CD19-directed CAR T cells have shown remarkable anti-cancer activity in patients with B-cell malignancies. In total, the CAR-T cells have demonstrated fabulous clinical responses in the treatment of mostly relapsed or refractory hematological malignancies that led to four FDA-approved CAR T cell therapy including Kymriah, Yescarta, Tecartus, and Breyanzi over the past 4 years [42–44]. Despite the significant success in the treatment of malignancies, CAR T cell therapy has shown several challenges. First, treatment with CAR T cells can cause serious side effects, such as cytokine release syndrome (CRS) and immune effector cell-associated neurotoxicity syndrome (ICANS) [45]. Second, the autologous CAR T cell-manufacturing process can be long, costly, and laborious. Based on these limitations, other subsets of immune cells, such as natural killer (NK) cells, have received more attention in immunotherapy [46].

Up to now, 19 clinical trials involving CAR NK cells have been reported for the treatment of malignancies [42]. It seems that CAR NK cells can act as an alternative therapy option to CAR-T cells due to an intrinsic killing capacity of malignant cells and only a few adverse events related to insertional mutagenesis. CRS typically develops after CAR T cell administration due to elevated proinflammatory cytokines, such as IL-1, IL-6, and TNFα [47].

Notably, CAR NK cells are considered safe because they mainly secrete IFN-γ and GMCSF. In addition, the approved CAR T cell products have been autologous through the risk of graft-versus-host disease (GVHD) with the use of allogeneic T cells. Several clinical studies have shown that, unlike allogeneic T cells, allogeneic NK cells do not cause GVHD [48].

According to pre-clinical and clinical studies, CAR NK cell therapy is a considerable anti-cancer agent and is safer than CAR-T cell therapy [46]. Nonetheless, CAR-NK cell therapy encounters some limitations, such as the expansion and activation of primary NK cells in vitro, the hardness of storing and shipping NK cell products (due to the sensitivity of NK cells to cryopreservation), and the low transduction efficiency. Therefore, further research is still required to optimize CAR NK cell therapy [39, 42].

### Combining oncolytic viruses with CAR T cell therapy

Chimeric antigen receptor (CAR) T cell therapy has generated significant excitement in managing hematological malignancies, but solid tumors pose various challenges. It seems that combinations of different targeted therapies may be required to attain efficient and complete responses in solid tumors [49]. Oncolytic viruses as potential anti-tumor agents can help CAR T cells simultaneously overcome some of the limitations found in solid tumors [21].

Generally, the anti-tumor function of tumor-targeting CD8 + T-cells depends on three signals, including TCR engagement, co-stimulation, and an inflammatory stimulus. The second-and third-generation CARs provide engineered T-cells with TCR engagement and co-stimulation signaling [50]. The inflammatory stimulation of CAR T cells is typically driven by cytokines like IL-12 or type I IFNs. Type I IFNs are essential natural mediators of antiviral and anti-tumor activity by stimulating host adaptive immunity. It is identified that OVs can induce an elevated type I IFN signature in the tumor microenvironment [51].

In addition, many solid tumors intervene with tumor-infiltrating immune cells by diminishing adhesion molecule expression on endothelium, decreasing chemokine production, and secreting extracellular matrix components to prevent T cells from reaching the tumor microenvironment [52]. As mentioned, impaired CAR T-cell trafficking into solid tumors is one of the main challenges faced in applying CAR T cell therapy. Previous studies has shown that an inflammatory tumor microenvironment with active dendritic cells promotes type I IFNs and IFN-γ secretion, and moreover co-stimulatory signals are essential for CAR T cell infiltration. Therefore, CAR T-cells are likely to benefit from the synergistic combination with OVs that enhanced CAR T cell migration into the tumor and persistence within the tumor microenvironment [51, 53].

The upregulation of programmed death ligand-1 (PD-L1) on cancer cells negatively regulates T cell function and contributes to cancer immune escape [54]. On the other hand, cancer cells also induce immunosuppressive signaling mediated by a variety of immune cells like Tregs, tumor-associated macrophages (TAMs), myeloid-derived suppressor cells (MDSCs), fibroblasts, and endothelial cells within the tumor microenvironment. These immunosuppressive phenotypes impede effector T-cell exclusion and function within the tumor microenvironment. Overcoming these mechanisms of immunosuppression can improve the efficacy of immunotherapy, especially CAR T cell therapy in solid tumors. It seems that the combination of OVs and CAR-T cells can negate the multiple immunosuppressive mechanisms and enhance the effector functions of T cells [52, 55].

OVs have recently been generated by genetic modification to have co-stimulatory molecules including OX40L, 4-1BBL, and GITRL to boost the local activation and expansion of effector T cells within the tumor microenvironment [51]. Moreover, OVs have been designed with additional transgenes encoding CD40 ligand (CD40L), increasing local CD40 activation within the tumor microenvironment. Owing to the expression of CD40 on a wide range of immune cells, including CD4^+^ T-cells, macrophages, and B-cells, activation of this pathway mediates anti-tumor *immune* responses [56].

**Fig. 1:**
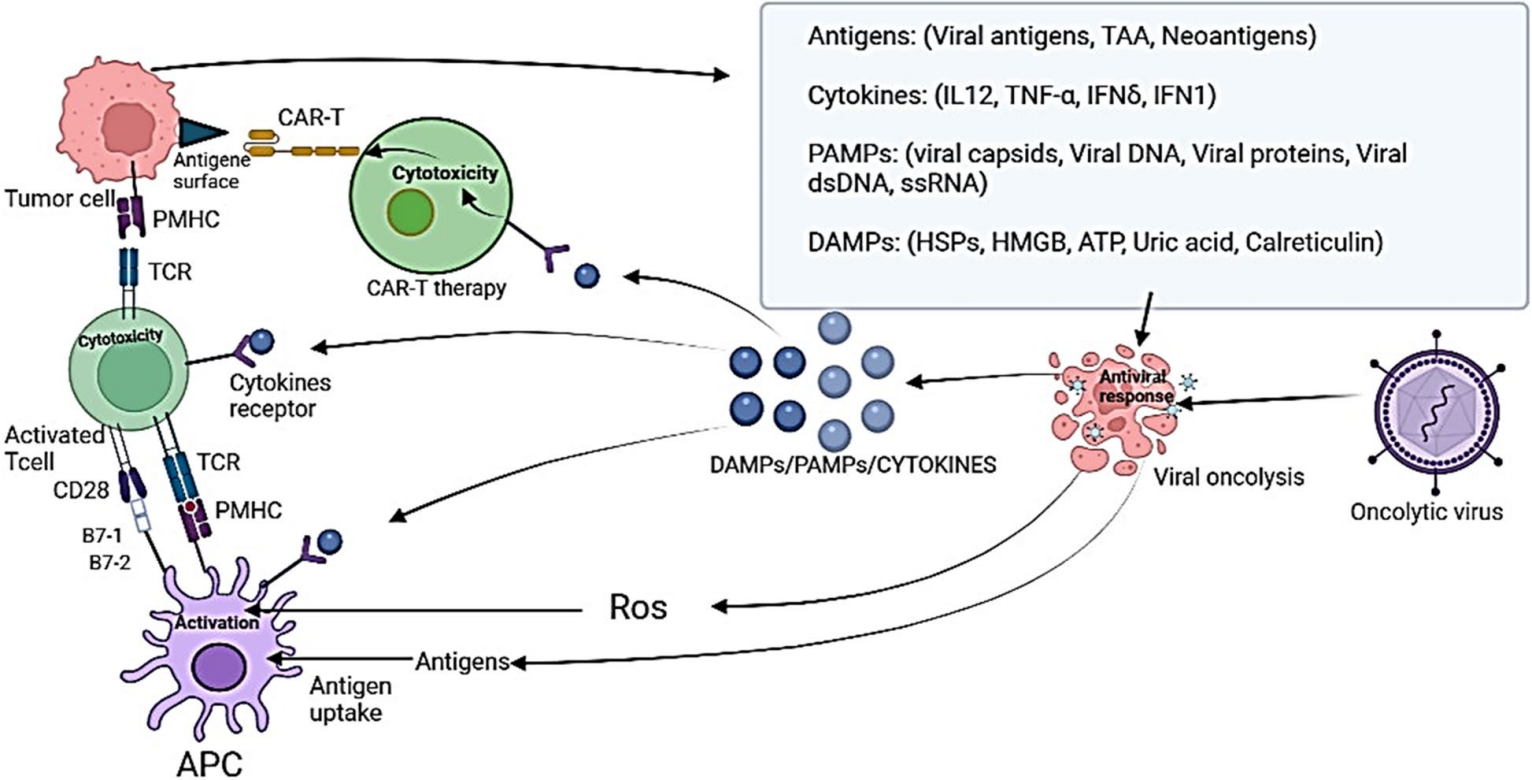
The anti-tumor impacts of CAR-T cells in combination with engineered-OVs.

Various pre-clinical research has evaluated different transgene-armed OV in combination with CAR-T cells [51]. The finding of these researches gave main insights into the anti-tumor impacts of CAR-T cells in combination with engineered-OVs (Fig. 1). Furthermore, the flexibility of the engineering process of OVs and CAR T cells can help researchers design the most appropriate combinations of recombinant OV and CAR T cell co-stimulation to the specific characteristics of the targeted tumor [57].

**Fig. 2:**
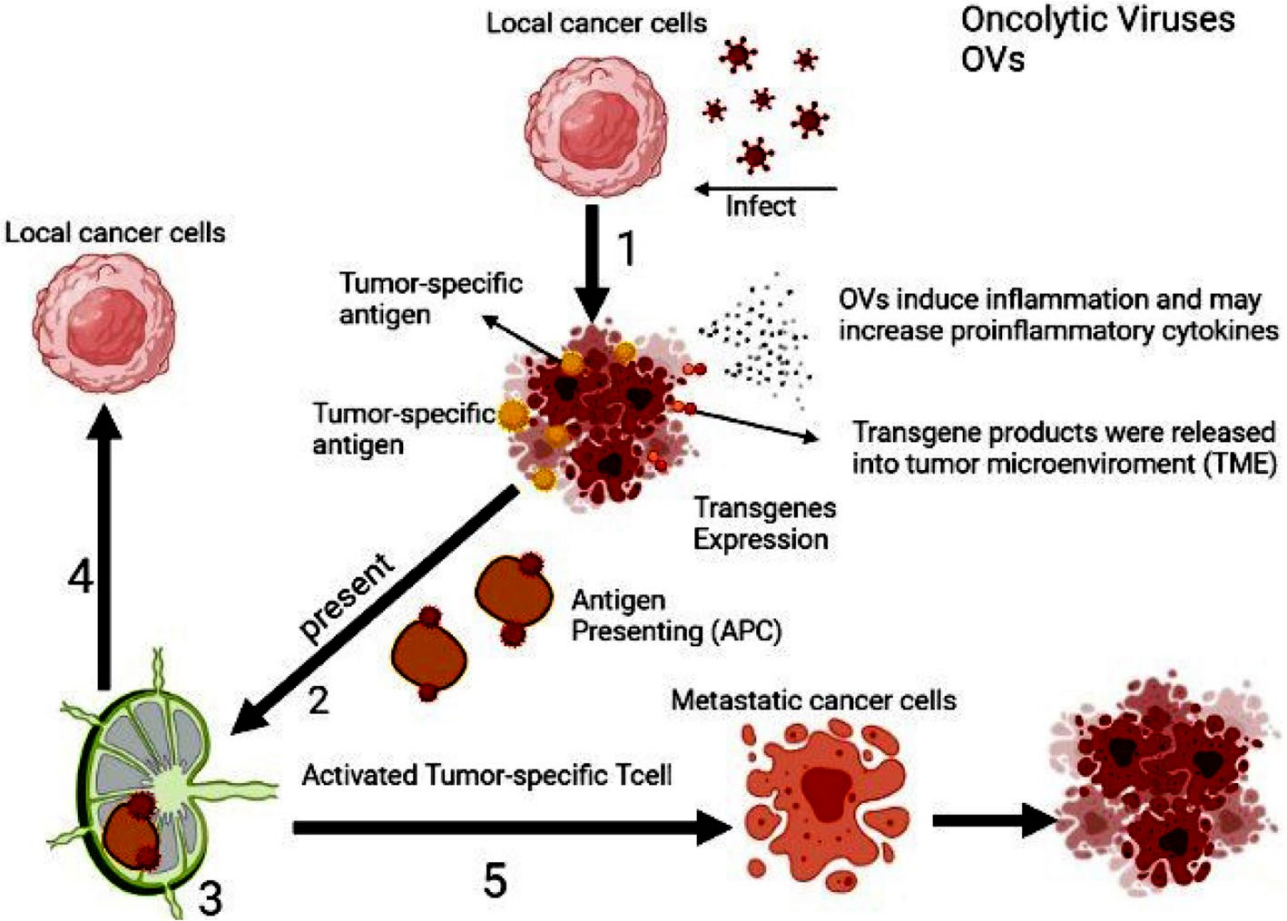
Different levels of combined therapy of OVs with CAR-T cells. Oncolytic viruses exert their effects on cancer cells in a variety of ways. These viruses can lysis cancer cells to better activate APC cells, cause inflammation, and release inflammatory cytokines

In fact, the immune-stimulatory properties of OVs and the potential to arm OVs with therapeutic transgenes make them excellent partners to boost CAR T cells in vivo (Fig. 2). Nonetheless, the potential for combining OVs, CAR T-cell therapy, and an additional immunotherapeutic strategy are practically limitless [58]. Naturally, translation of pre-clinical experimental results to clinical trials cannot often be logical and acceptable because of using an immunodeficient mouse model (NOD SCID gamma mice), so this experimental system cannot model the interactions between OV and CAR T cells in the human immune system [59]. In addition, the double combination enhances concern about the safety of combining two potent proinflammatory immunotherapies. Finally, given that combining these two therapies is a new approach, many studies are needed to improve it.

### Use of oncolytic viruses in combination with CAR-T cell therapy in pre-clinical and clinical studies

Scientist’s achievements in recombinant genetic engineering sciences have led to significant progress in oncolytic viruses’ usage. By oncolytic viruses’ genome editing, the ability of these viruses to destroy the tumor cells can be altered, and their oncotropic nature can be improved. Inserting the oncolytic viruses into the target cells could turn the cancer cells into cytokine and chemokine factories, thus modifying TME (tumor microenvironment) from an immunosuppressive environment to an immunostimulatory one to facilitate the summoning and induction of the immune cells (such as CAR-T cells) and factors [51]. Tumor cells will try to suppress the immune system, but after contaminating with oncolytic viruses, danger signals are sent, and therefore immune cells such as CD8 + T cells will arrive at the scene and destroy the tumor cells [60]. Until now, many manipulated oncolytic viruses have been used in pre-clinical and clinical steps of researches. The results of using oncolytic viruses and CAR-T cell therapy spontaneously in animal studies have been promising. In the following, we discuss the results of the clinical and pre-clinical studies about CAR-T cell therapy combined with oncolytic viruses.

In a study conducted by Anna Wing et al. on pancreatic ductal carcinoma/colorectal carcinoma, Onc. Ad-EGFR BITE adenovirus, which is armed by EGFR-targeting, a bispecific T-cell engager was used. Concomitant use of this engineered virus and the CAR-T cells containing 4-1BB endodomain and targeting folate receptor alpha (FR-α) antigen will improve the function of the CAR-T cells. This happens because of the BITE secretion of the contaminated cancer cells. Although tumor cells are free of CAR-specific antigens (FR-α in here) but can be targeted and destroyed by the CAR-T cells thanks to BITE secretion, and this is where BITE importance is two folded [19]. Pancreatic ductal carcinoma has a highly immunosuppressive TME and increased Treg cells. So pancreatic tumors TME can disturb T-cell function [61, 62]. In a study relevant to this malignancy, they utilized the TNF-α and IL-2 expressing Onc.Ad-TNF-α/IL-2 oncolytic virus combining with Mesotheline (meso)-specific CAR-T cells containing 4–1 BB endodomain improved the efficiency of the anti-tumor activity of the CAR-T cells (in comparison with using it alone) in mouse models. This improvement in the efficiency of the CAR-T cells could be related to the local expression of the TNF-α and IL-2 in addition to the oncolysis characteristics of these viruses [63]. These cytokines could expand the number of tumor-infiltrating cells in the tumor microenvironment [63, 64].

Efficient animal studies have been performed in the combined oncolytic viruses and CAR-T cell therapy to treat other carcinomas such as head and neck carcinoma and neuroblastoma. Using of the Onc. Ad5Δ24 virus (armed with RANTES chemokine and IL-15 cytokine) along with Ganglioside GD2-specific CAR-T cells to treat the tumors leads to an increase in the attraction and survival of the CAR-T cells in neuroblastoma tumor environment [65]. In research performed by Amanda Rosewell Shaw et al. on head and neck cancer metastasis, a type of binary oncolytic adenovirus named CAdVECIL12p70/αPDL1 was employed in combination with human epidermal growth factor 2 (HER2)-specific CAR-T cells. The construct used in this study encoded the PD-L1 blocking antibody concomitantly with IL-12P70. Local production of the anti-PD-L1 in the tumor site is the advantage of this oncolytic virus [66]. Systemic applying the antibodies against the immune checkpoint blockers like PD-L1 and CTLA-4 can lead to unwanted manifestations such as autoimmunity and even tumor growth. Therefore, it appears that manipulated oncolytic viruses could reduce the inappropriate systemic effects of these treatments by local production of these antibodies [67, 68].

Oncolytic viruses can cooperate with lymphocytes against malignancies, but because there is an immune response against these viruses, therapeutic doses of them to dominate the immune responses, including antibodies, are very high [69, 70]. To overcome this issue, Heather VanSeggelen et al. investigated using CAR-T cells loaded with low-dose oncolytic viruses. The results of this study disclosed that there is no difference in the CAR expression inside virus-loaded CAR-T cells with intact CAR-T cells, and there was no disturbance in the function of manipulated CAR-T cells. It can be said that CAR-T cells successfully transferred the virus to tumor cells [71]. Although this was an in vitro study, the results are auspicious since combining two treatment strategies comes to meaning only when there is no interference between the treatments. According to the results of this study, utilizing the “variable oncolytic virus-loaded CAR-T cells” can be suggested to future pre-clinical researches and survey the results of this novel method in animal models of cancer.

Many clinical trials about the use of oncolytic viruses in tumor treatment are on the line. In 2015, the FDA approved the talimogene laherparepvec (T-VEC) license for melanoma. T-VEC is an engineered HSV-1, which expresses human granulocyte–macrophage colony-stimulating factor (GM-CSF). GM-CSF increases the summoning of the antigen-presenting cells to the tumor environment. T-VEC provokes tumor death with specific proliferation in the tumor cell and stimulates the specific immune system response against the tumor as a secondary function [72]. Applying T-VEC as a combination with chemotherapy [73], radiotherapy [74], and drugs like ipilimumab (anti-cytotoxic T lymphocyte antigen 4 antibody) [75] are available approaches to increase the efficiency of this therapeutic method. Therefore, considering these cases, the T-VEC and CAR T-cell combination can be investigated in the future. However, only one approved clinical trial investigated CAR T and OV combination therapy for HER2-positive tumors (NCT03740256) [76]. Selecting a particular combination of the oncolytic virus and a CAR vector relates to various items, such as the virus’s ability to contaminate cells that are expressing CAR antigen.

## Conclusion

Tumor and their treatment have been one of the challenges in medical science until now. The FDA approval of two CAR-T cells against hematologic malignancies, tisagenlecleucel-T (Kymriah, Novartis) and axicabtagene ciloleucel (YesCAR Ta, Kite Phama), as well as the first OV (talimogene laherparepvec) for the treatment of melanoma, all are inspiring and significant advances in the field of cancer immunotherapy for the control and cure of an incurable disease. However, issues like possible pathogenicity of viable biologic agents, virus elimination by the immune system, not so high efficacy of CAR-T cell therapy in treating solid tumors, CAR-T cell-induced cytokine storms, and the same obstacles are very complex challenges in the road of clinical development. This review will discuss two cancer treatment approaches: CAR-T cell therapy and using the oncolytic viruses and combination use of them. It is demonstrated in pre-clinical studies and various animal models of cancer that using these treatments concomitantly is more efficient than using them alone. However, further investigations in this field to determine the appropriate virus, proper administration method, and finding possible complications are highly suggested. The combined use of CAR-T cell therapy and the oncolytic viruses can reduce the evading of the virus from immunotherapy. These discoveries enclose that this combined method can cause remarkable evolution in cancer treatment, especially solid tumors.

## Data Availability

The data achieved and analyzed during this study are available from the corresponding author on reasonable request.

## Abbreviations

PMHC: Peptide-major histocompatibility complex
TAA: Tumor-associated antigen
PAMP: Pathogen-associated molecular pattern
DAMP: Damage-associated molecular pattern’
HSPs: Heat shock proteins
